# Incidence of Herpes Simplex Virus Type 2 Infection Among African Women Using Depot Medroxyprogesterone Acetate, a Copper Intrauterine Device, or a Levonorgestrel Implant for Contraception: A Nested Randomized Trial^[Author-notes ciab1027-FM1]^

**DOI:** 10.1093/cid/ciab1027

**Published:** 2021-12-15

**Authors:** Nelly R Mugo, Randy M Stalter, Renee Heffron, Helen Rees, Caitlin W Scoville, Charles Morrison, Athena P Kourtis, Elizabeth Bukusi, Mags Beksinska, Neena M Philip, Ivana Beesham, Jen Deese, Vinodh Edward, Deborah Donnell, Jared M Baeten, Evidence for Contraceptive Options and HIV Outcomes (ECHO) Trial Consortium, Jared M Baeten, James Kiarie, Timothy D Mastro, Nelly R Mugo, Helen Rees, Jessica Justman, Zelda Nhlabatsi, Elizabeth A Bukusi, Maricianah Onono, Cheryl Louw, Linda Gail Bekker, Gonasagrie Nair, Mags Beksinska, Jennifer Smit, G Justus Hofmeyr, Mandisa Singata-Madliki, Jennifer Smit, Thesla Palanee-Phillips, Raesibe Agnes Pearl Selepe, Sydney Sibiya, Khatija Ahmed, Margaret Phiri Kasaro, Jeffrey Stringer, Deborah Baron, Deborah Donnell, Peter B Gichangi, Kate B Heller, Nomthandazo Mbandazayo, Charles S Morrison, Kavita Nanda, Melanie Pleaner, Caitlin W Scoville, Kathleen Shears, Petrus S Steyn, Douglas Taylor, Katherine K Thomas, Julia D Welch

**Affiliations:** Center for Clinical Research & Center for Microbiology Kenya Medical Research Institute (KEMRI), Kenya; Department of Global Health, Seattle, Washington, USA; Department of Global Health, Seattle, Washington, USA; Department of Epidemiology, University of Washington, Seattle, Washington, USA; Department of Global Health, Seattle, Washington, USA; Department of Epidemiology, University of Washington, Seattle, Washington, USA; University of the Witwatersrand, Wits Reproductive Health and HIV Institute (Wits RHI), Johannesburg, South Africa; Department of Global Health, Seattle, Washington, USA; Behavioral, Epidemiologic and Clinical Sciences, FHI 360, Durham, North Carolina, USA; Division of HIV Prevention, National Center for HIV, Hepatitis, STD and TB Prevention, Centers for Disease Control and Prevention, Atlanta, Georgia, USA; Center for Clinical Research & Center for Microbiology Kenya Medical Research Institute (KEMRI), Kenya; Department of Global Health, Seattle, Washington, USA; MatCH Research Unit (MRU), Department of Obstetrics and Gynaecology, Faculty of Health Sciences, University of the Witwatersrand, Durban, South Africa; ICAP at Columbia University, Mailman School of Public Health, Columbia University, New York, New York, USA; MatCH Research Unit (MRU), Department of Obstetrics and Gynaecology, Faculty of Health Sciences, University of the Witwatersrand, Durban, South Africa; Department of Medicine, University of Washington, Seattle, Washington, USA; The Aurum Institute, Johannesburg, South Africa; School of Pathology, Faculty of Health Sciences, University of the Witwatersrand, Johannesburg, South Africa; Vaccine and Infectious Disease Division, Fred Hutchinson, Seattle, Washington, USA; Department of Global Health, Seattle, Washington, USA; Department of Epidemiology, University of Washington, Seattle, Washington, USA; Department of Medicine, University of Washington, Seattle, Washington, USA

**Keywords:** herpes simplex virus type 2 (HSV-2), HIV, contraception, women, Africa

## Abstract

**Background:**

Globally, women have higher herpes simplex virus type 2 (HSV-2) prevalence than men; data from observational studies suggest a possible association of HSV-2 acquisition with use of intramuscular depot medroxyprogesterone acetate (DMPA-IM).

**Methods:**

Within a randomized trial of the effect of 3 contraceptive methods—DMPA-IM, a copper intrauterine device (IUD), and a levonorgestrel (LNG) implant—on human immunodeficiency virus (HIV) acquisition, we assessed HSV-2 acquisition. HSV-2 and HIV seronegative women, aged 16–35 years, and seeking effective contraception were followed for 12–18 months at 12 sites in Eswatini, Kenya, South Africa, and Zambia from 2015 to 2018. HSV-2 serologic testing was done at enrollment and final study visits. Intention-to-treat analysis using Poisson regression with robust standard errors compared HSV-2 incidence by contraceptive method.

**Results:**

At baseline, 4062 randomized women were HSV-2 seronegative, of whom 3898 (96.0%) had a conclusive HSV-2 result at their final study visit. Of these, 614 (15.8%) acquired HSV-2, at an incidence of 12.4/100 person-years (p-y): 10.9/100 p-y among women assigned DMPA-IM, 13.7/100 p-y the copper IUD, and 12.7/100 p-y the LNG implant. Incidence rate ratios (IRR) for HSV-2 acquisition were 0.80 (95% confidence interval [CI], .65–.97) for DMPA-IM compared with copper IUD, 0.86 (95% CI, .71–1.05) for DMPA-IM compared with LNG implant, and 1.08 (95% CI, .89–1.30) for copper IUD compared with LNG implant. HSV-2 acquisition risk was significantly increased among women who also acquired HIV during follow-up (IRR 3.55; 95% CI, 2.78–4.48).

**Conclusions:**

In a randomized trial, we found no association between HSV-2 acquisition and use of 3 contraceptive methods.

**Trial registration:**

ClinicalTrials.gov number NCT02550067.

## Teaser

Data from observational studies suggest a possible association of herpes simplex virus type 2 (HSV-2) acquisition with use of intramuscular depot medroxyprogesterone acetate (DMPA-IM). In a randomized trial, we found no association between HSV-2 acquisition and use of 3 contraceptive methods.

An estimated half a billion persons globally are infected with herpes simplex virus type 2 (HSV-2) infection, of whom 64% are women [1]. There is strong and well-established epidemiological evidence of synergy between HSV-2 infection and risk of both human immunodeficiency virus (HIV) acquisition and transmission, with prevalent HSV-2 increasing HIV risk 3-fold in women and incident HSV-2 increasing HIV risk by an even greater factor [2–5]. Though commonly not life-threatening, HSV-2 infection nevertheless has important adverse health consequences, including recurrent genital ulcer disease, stigma and adverse social consequences, and the potential for perinatal transmission to newborns and associated significant sequelae [6].

In African populations, both HIV and HSV-2 disproportionately affect women. Considerable global attention has focused on whether use of certain contraceptive methods increases the risk of acquisition of HIV. Fewer data have assessed whether contraceptive method influences acquisition of HSV-2. Three prospective observational studies, among sex workers in Canada [7] and Rwanda [8] and among a general population cohort in Uganda [9], found an increased risk of HSV-2 acquisition among women using the injectable contraceptive depot medroxyprogesterone acetate delivered intramuscularly (DMPA-IM). Laboratory studies have suggested that DMPA-IM exposure could decrease cell–cell adhesion and mucosal barrier function, providing a potential mechanism for increased HSV-2 susceptibility [10]. In contrast, one observational study among sex workers in Kenya [11] and another among HIV-serodiscordant couples in east and southern Africa [12] found no elevated risk for HSV-2 acquisition with use of hormonal contraception.

We conducted a randomized, open-label clinical trial of the effect of 3 commonly used contraceptive methods: DMPA-IM, a copper intrauterine device (IUD), and a levonorgestrel (LNG) implant on HIV incidence [13]. In this secondary analysis, we assess the impact of contraceptive method on acquisition of HSV-2.

## METHODS

### Study Population and Procedures

Between December 2015 and October 2018, 7829 HIV seronegative women aged 16–35 years were enrolled and followed at 12 clinical trial sites in 4 African countries: Eswatini, Kenya, South Africa, and Zambia. As previously reported, the trial did not find substantial difference in HIV risk among users of the contraceptive methods evaluated, and all methods were safe and highly effective for pregnancy prevention [13]. Women were recruited from family planning or reproductive health clinics serving postpartum and postabortion clients, other relevant clinics, and the general community. Eligible participants were women aged 16–35 years, who were seeking effective contraception, were sexually active, had no medical contraindications to the trial contraceptive methods, were not pregnant, were willing to be randomized to 1 of the 3 study contraceptive methods, agreed to use the assigned method for 18 months, and reported not using injectable, intrauterine, or implantable contraception for the 6 months before enrollment.

At enrollment, women were assigned in a 1:1:1 ratio to receive either DMPA-IM (150 mg/1 mL, Depo Provera, Pfizer, every 13 weeks), copper IUD (Optima TCu380A, Injeflex), or LNG implant (Jadelle, Bayer), using variable block size randomization stratified by site. Contraceptives were provided on site, and confirmation of placement was ascertained at each visit for LNG implant, and for copper IUD at month 1, as clinically indicated, and final visit. Women were assessed 1 month after enrollment to address initial contraceptive side effects, following which, participants attended a visit at month 3 and then quarterly for scheduled study visits. Women were counseled that they had the option to continue, change, or stop using their assigned study contraceptive method at any time during study follow-up. Women discontinuing their study contraceptive method remained in the trial. Visit procedures included HIV serological testing and contraceptive counseling. Assessment of sexual behavior was done quarterly. Participants received comprehensive HIV prevention services including HIV risk-reduction counseling, quarterly syndromic sexually transmitted infection management, provision of free condoms, and offer of HIV preexposure prophylaxis (PrEP) as per national guidelines [14]. Women who acquired HIV continued in the study and were referred to local facilities for HIV care. Follow-up was up to 18 months, depending on calendar time of enrollment into the trial, with later enrollees followed for 12 or 15 months.

HIV counseling and testing was done at screening, enrollment, and quarterly study visits. Testing of genital swabs for evaluation of *Neisseria gonorrhoeae* and *Chlamydia trachomatis* was done at the enrollment and final study visits. Pregnancy testing was done at enrollment and final visits and when clinically indicated.

### HSV-2 Assessment

Serum samples archived at enrollment and final study visits were analyzed for HSV-2 following a standard algorithm (Supplemental Figure 1). In cases in which serum from the participant’s final study visit was not available, the first available sample collected before the final visit was analyzed. Baseline samples were first tested using the HerpeSelect 2 ELISA IgG (Focus Diagnostics, Cypress Hill, CA, USA). Women with enzyme-linked immunosorbent assay (ELISA) index values > 3.5 were considered positive and not tested further, whereas those with index values < 0.90 were considered negative and those with index values ≥ 0.90 or ≤ 3.50 were considered equivocal [15]. Women with negative or equivocal baseline results had their final visit sample tested. An index value < 0.90 at exit was considered HSV-2 negative, and women were considered to have not acquired HSV-2 during follow-up. Those with final visit samples with index values ≥ 0.90 (equivocal or positive) were considered to potentially have acquired HSV-2 during follow-up. To confirm HSV-2 acquisition, further testing was done using HSV-2-specific Western blot analysis, performed by the University of Washington Virology Laboratory (Seattle, WA, USA) [16]. Women with equivocal baseline ELISA results underwent Western blot testing of both baseline and final visit samples, whereas women with negative baseline ELISA results had Western blot performed on their final visit sample only. For those with final visit Western blot results that were indeterminate, further Western blot testing of paired baseline and exit visit samples was performed. Women with a negative baseline result and positive Western blot at the final visit were considered to have incident HSV-2 infection. Women who had HIV seroconversion during study follow-up had serum tested for HSV-2 at that visit as well as at their final study visit.

### Ethics Statement

Ethics review committees at each study site approved the study protocol. All participants provided written informed consent, which included counseling about randomization, each study contraceptive method, and their rights as research participants.

### Statistical Analysis

This analysis was restricted to the subset of women confirmed to be HSV-2 negative at enrollment and who had a definitive final visit HSV-2 result (i.e., positive or negative). Descriptive analyses were used to present characteristics of the population, compared by randomized contraceptive group. The primary endpoint was incident HSV-2, defined as having a confirmed negative HSV-2 result at enrollment and a positive final study visit result. For women who acquired HIV during follow-up, the final study visit, rather than the visit after which HIV was acquired, was used to avoid potential bias associated with truncating follow-up correlated with HIV acquisition and attendant higher sexual risk behaviors.

To assess the effect of contraceptive method on HSV-2 acquisition, we carried out both intention-to-treat and best-achievable-use analyses. The best-achievable-use analysis was restricted to women who began use of their assigned method at enrollment (or within 28 days of enrollment for copper IUD users) and had continuous use up to the date of their final visit HSV-2 test. For both analyses, Poisson regression with robust standard errors was used. Models were adjusted for country and included an offset for follow-up time. This was calculated as the time between the enrollment and final visit HSV-2 test dates for women who did not acquire HSV-2. For women who did acquire HSV-2, method exposure was calculated as the midpoint between the baseline and final visit HSV-2 test result dates. We report the incidence rate ratio (IRR) estimate with 95% confidence interval (CI). Subgroup analyses based on age (< or ≥ 25 years) and region (Eswatini and South Africa/Kenya and Zambia) were conducted and the IRR and 95% CI were estimated for each subgroup; the interaction between randomization arm and each subgroup variable was assessed.

In an additional analysis, we assessed factors correlated with HSV-2 acquisition, using Poisson regression with robust standard errors; factors assessed were specific baseline and final visit sexual risk behaviors, incident HIV infection, *C trachomatis*, and *N gonorrhoeae* (baseline and final visit). Time was added to models as an offset. All models included randomized assignment and country. Two multivariate models were constructed: in the first model, all variables individually achieving a *P* value < .20 were included. In the second, covariates were removed from the first model to achieve the best fit using the Akaike information criterion.

All analyses were conducted using SAS version 9.4 (SAS Institute Inc., Cary, NC, USA) and R version 3.6.1 (R Core Team, Vienna, Austria).

## RESULTS

### Study Participants

**Figure 1.**
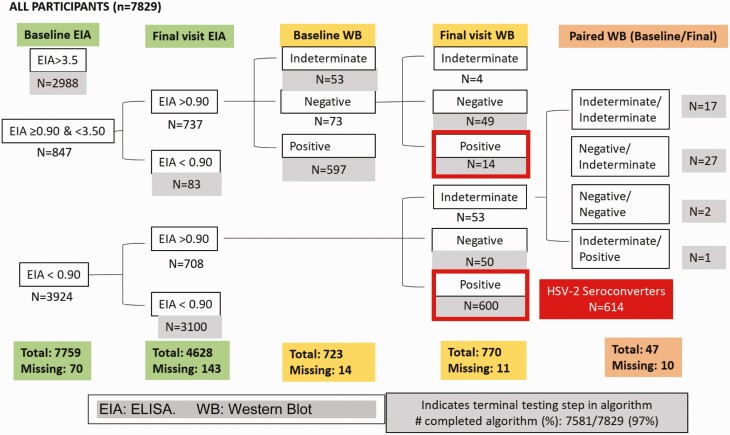
Herpes simplex virus type 2 testing algorithm. Abbreviations: EIA, ELISA; HSV-2, herpes simplex virus type 2; WB, western blot.

**Figure 2.**
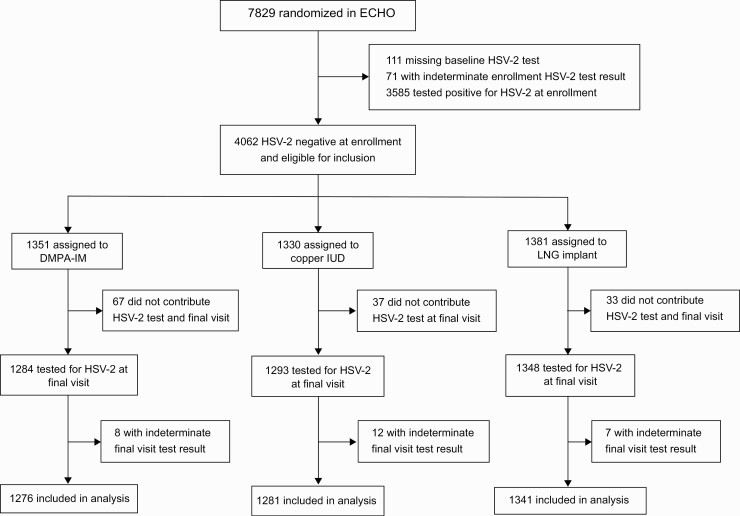
CONSORT diagram. Abbreviations: HSV-2, herpes simplex virus type 2; IUD, intrauterine device; LNG, levonorgestrel.

Of 7829 HIV seronegative women enrolled in the Evidence for Contraceptive Options and HIV Outcomes trial, 7759 (99.1%) were assessed by HSV-2 ELISA at baseline (Figure 1), of whom 2988 (38.5%) were HSV-2 seropositive. Among 4771 with HSV-2 negative or indeterminate baseline results, 4628 (97.0%) had a final visit sample available for HSV-2 ELISA testing; 1426 of these 4628 women (30.8%) underwent HSV-2 Western blot testing because of an indeterminate or positive ELISA result. In total, 4062 women were classified as HSV-2 seronegative at baseline (Figure 2). Of these, 3898 (96.0%) had a conclusive final visit result and contributed to the final analyses: 1276 were assigned DMPA-IM, 1281 copper IUD, and 1341 LNG implant.

Three-quarters were < 25 years of age, 81% had never married, approximately one-quarter lived with a partner, approximately three-quarters had a previous pregnancy, and 91% had at least some secondary school education (Table 1). Few (1%) reported receiving money or gifts for sex, 4% reported a new sex partner, and 71% reported condomless sex acts in the 3 months before enrollment. Baseline characteristics of enrolled women were similar across randomization groups (Tables 2, 3).

**Table 1.** Baseline Characteristics

	DMPA-IM Group (N = 1351)	Copper IUD Group (N = 1330)	LNG Implant Group (N = 1381)	Total (N = 4062)
	n (%) or Median (IQR)	n (%) or Median (IQR)	n (%) or Median (IQR)	n (%) or Median (IQR)
Age, y				
16–17	10 (0.7%)	17 (1.3%)	15 (1.1%)	42 (1.0%)
18–20	481 (35.6%)	466 (35.0%)	478 (34.6%)	1425 (35.1%)
21–24	526 (38.9%)	516 (38.8%)	542 (39.2%)	1584 (39.0%)
25–30	264 (19.5%)	272 (20.5%)	295 (21.4%)	831 (20.5%)
31–35	70 (5.2%)	59 (4.4%)	51 (3.7%)	180 (4.4%)
Marital status				
Never married	1092 (80.8%)	1072 (80.6%)	1106 (80.1%)	3270 (80.5%)
Married	253 (18.7%)	255 (19.2%)	267 (19.3%)	775 (19.1%)
Previously married	6 (0.4%)	3 (0.2%)	8 (0.6%)	17 (0.4%)
Lives with partner	377 (27.9%)	346 (26.0%)	375 (27.2%)	1098 (27.0%)
Education				
No schooling	4 (0.3%)	5 (0.4%)	9 (0.7%)	18 (0.4%)
Primary school	93 (6.9%)	105 (7.9%)	130 (9.4%)	328 (8.1%)
Secondary school	1013 (75.0%)	983 (73.9%)	1000 (72.4%)	2996 (73.8%)
Postsecondary school	241 (17.8%)	237 (17.8%)	242 (17.5%)	720 (17.7%)
Any previous pregnancy	998 (73.9%)	1009 (75.9%)	1053 (76.2%)	3060 (75.3%)
Body mass index > 30 kg/m2	265 (19.6%)	271 (20.4%)	317 (23.0%)	853 (21.0%)
More than 1 sex partner, prior 3 mo	74 (5.5%)	79 (5.9%)	72 (5.2%)	225 (5.5%)
New sex partner, prior 3 mo	49 (3.6%)	62 (4.7%)	47 (3.4%)	158 (3.9%)
Number of vaginal sex acts, prior 3 mo	8.0 (4.0, 18.0)	8.0 (3.0, 20.0)	7.0 (3.0, 18.0)	8.0 (3.0, 18.0)
Any condomless sex, prior 3 mo	945 (70.0%)	961 (72.3%)	984 (71.3%)	2890 (71.2%)
No condom last vaginal sex	703 (52.1%)	660 (49.6%)	696 (50.4%)	2059 (50.7%)
Sex for money or gifts, prior 3 mo	12 (0.9%)	11 (0.8%)	11 (0.8%)	34 (0.8%)
*Chlamydia trachomatis* infection	236 (17.5%)	253 (19.0%)	245 (17.7%)	734 (18.1%)
*Neisseria gonorrhoeae* infection	49 (3.6%)	53 (4.0%)	51 (3.7%)	153 (3.8%)

Abbreviations: DMPA-IM, intramuscular depot medroxyprogesterone acetate; IQR, interquartile range; IUD, intrauterine device; LNG, levonorgestrel.

**Table 2.** Comparison of HSV-2 Incidence Among DMPA-IM, Copper IUD, and LNG Implant Users—Intention-to-treat Analysis

	DMPA-IM Group	Copper IUD Group	LNG Implant Group	DMPA-IM vs Copper IUD	DMPA-IM vs LNG Implant	Copper IUD vs LNG Implant
	Events/p-y	Events/p-y	Events/p-y	IRR (95% CI), *P* Value	IRR (95% CI), *P* Value	IRR (95% CI), *P* Value
Overall	178/1631 (10.9/100 p-y)	221/1614 (13.7/100 p-y)	215/1693 (12.7/100 p-y)	0.80 (0.65–0.97), *P* = .02	0.86 (0.71–1.05), *P* = .1	1.08 (0.89–1.30), *P* = .4
Age category						
Age < 25 y	140/1219 (11.5/100 p-y)	178/1195 (14.9/100 p-y)	171/1261 (13.6/100 p-y)	0.78 (0.62–0.97), *P* = .02	0.85 (0.68–1.07), *P* = .2	1.10 (0.89–1.36), *P* = .4
Age 25 + y	38/412 (9.2/100 p-y)	43/418 (10.3/100 p-y)	44/432 (10.2/100 p-y)	0.92 (0.59–1.42), *P* = .7	0.92 (0.59–1.42), *P* = .7	1.00 (0.66–1.52), *P* = .99
Age interaction				*P* = .5	*P* = .8	*P* = .7
Site location^a^						
Eswatini and South Africa sites	163/1280 (12.7/100 p-y)	187/1282 (14.6/100 p-y)	191/1338 (14.3/100 p-y)	0.87 (0.71–1.08), *P* = .2	0.89 (0.72–1.10), *P* = .3	1.02 (0.84–1.25), *P* = .8
Kenya and Zambia sites	15/350 (4.3/100 p-y)	34/332 (10.3/100 p-y)	24/355 (6.8/100 p-y)	0.42 (0.23–0.77), *P* = .005	0.63 (0.33–1.20), *P* = .2	1.52 (0.90–2.55), *P* = .1^b^
Site interaction				*P* = .02	*P* = .3	*P* = .2

Abbreviations: CI, confidence interval; DMPA-IM, Intramuscular depot medroxyprogesterone acetate; IRR, incidence rate ratio; IUD, intrauterine device; LNG, levonorgestrel; p-y, person-years.

All models adjusted for country.

Comparison of LNG implant vs copper IUD (i.e., inverse of copper IUD vs LNG implant): IRR 0.66 (95% CI, .39–1.11), *P* = .1.

**Table 3.** Comparison of HSV-2 Incidence Among DMPA-IM, Copper IUD, and LNG Implant Users—Best Achievable Use Analysis

	DMPA-IM Group	Copper IUD Group	LNG Implant Group	DMPA-IM vs Copper IUD	DMPA-IM vs LNG Implant	Copper IUD vs LNG Implant
	Events/p-y	Events/p-y	Events/p-y	IRR (95% CI), *P* Value	IRR (95% CI), *P* Value	IRR (95% CI), *P* Value
Overall	112/1140 (9.83/100 p-y)	181/1316 (13.75/100 p-y)	186/1503 (12.38/100 p-y)	0.73 (0.58–0.93), *P* = .009	0.81 (0.64–1.03), *P* = .08	1.11 (0.91–1.36), *P* = .3
Age category						
Age < 25 y	83/845 (9.82/100 p-y)	146/965 (15.13/100 p-y)	146/1104 (13.22/100 p-y)	0.67 (0.51–0.88), *P* = .003	0.77 (0.59–1.00), *P* = .05	1.15 (0.91–1.44), *P* = .2
Age 25+ y	29/295 (9.84/100 p-y)	35/351 (9.96/100 p-y)	40/399 (10.04/100 p-y)	1.02 (0.63–1.67), *P* = .935	1.01 (0.63–1.63), *P* = .967	0.99 (0.63–1.56), *P* = .964
Age interaction				*P* = .1	*P* = .3	*P* = .6
Site location^a^						
South Africa, Eswatini sites	103/848 (12.15/100 p-y)	150/1039 (14.44/100 p-y)	166/1192 (13.92/100 p-y)	0.84 (0.66–1.08), *P* = .2	0.87 (0.68–1.11), *P* = .3	1.04 (0.83–1.29), *P* = .7
Kenya, Zambia sites	9/291 (3.09/100 p-y)	31/277 (11.18/100 p-y)	20/310 (6.45/100 p-y)	0.28 (0.13–0.58), *P* = .001	0.48 (0.22–1.05), *P* = .066	1.73 (0.99–3.03), *P* = .05^b^
Site interaction				*P* = .005	*P* = .2	*P* = .09

Abbreviations: CI, confidence interval; DMPA-IM, Intramuscular depot medroxyprogesterone acetate; IRR, incidence rate ratio; IUD, intrauterine device; LNG, levonorgestrel; p-y, person-years.

All models adjusted for country.

Comparison of LNG implant vs copper IUD (i.e., inverse of copper IUD vs LNG implant): IRR 0.58 (95% CI, .33–1.01), *P* = .05.

### Effect of Contraceptive Method Use and HSV-2 Acquisition

A total of 614 women acquired HSV-2 infection during study follow-up: 178 assigned DMPA-IM (incidence 10.9 per 100 person-years [p-y]), 221 assigned copper IUD (incidence 13.7 per 100 p-y), and 215 assigned the LNG implant (incidence 12.7 per 100 p-y). In intention-to-treat analysis (Table 2), there were no significant differences in HSV-2 incidence when comparing DMPA-IM versus LNG implant or copper IUD versus LNG implant. However, women assigned DMPA-IM had reduced HSV-2 incidence compared with those assigned a copper IUD, a result that was statistically significant (IRR 0.80; 95% CI, 0.65–0.97; *P* = .024).

Women aged < 25 years had higher HSV-2 incidence compared with women age ≥ 25 years across all contraceptive method groups, and the relationships between contraceptive method and HSV-2 incidence among women < 25 years were similar to the overall findings. HSV-2 incidence did not differ between DMPA-IM and copper IUD users among women ≥ 25 years of age, although the age interaction between women age < 25 and ≥ 25 years was not statistically significant (*P* = .53). HSV-2 incidence was also higher for all contraceptive methods among women in Eswatini and South Africa than among those from Kenya and Zambia. There was a significant interaction between region (Eswatini and South Africa/Kenya and Zambia sites) and randomization arm when comparing DMPA-IM users with copper IUD users (*P* = .024), and the DMPA-IM versus copper IUD relationship was only statistically significant for the Kenya and Zambia subgroup. Results of the best-achievable-use analyses (i.e., limited to women who remained on their assigned contraceptive at the final visit) were generally similar to those from the intention-to-treat analyses (Table 3).

### Correlates of HSV-2 Acquisition

HSV-2 acquisition was strongly associated with incident HIV infection (Table 4). Among 614 women who acquired HSV-2, there were 90 (14.7%) HIV infections compared with 79 (2.4%) among 3284 women who did not acquire HSV-2 (IRR 3.55; 95% CI, 2.78–4.48; *P* < .001). *N gonorrhoeae* at enrollment was also strongly associated with increased HSV-2 risk (IRR 1.44; 95% CI, 1.03–1.97; *P* = .026), as was *C trachomatis* (IRR 1.40; 95% CI, 1.15–1.69; *P* = .001) and *N gonorrhoeae* (IRR 1.87; 95% CI, 1.40–2.45; *P* < .001) infection at the final visit. Of the behavioral factors, living with a partner at baseline (IRR 0.64; 95% CI, .49–.82; *P* = .001) was associated with reduced risk for HSV-2 in the fully adjusted, multivariable, stepwise selection model.

**Table 4.** Baseline and Follow-up Factors Associated with HSV-2 Seroconversion

			Minimally Adjusted Models^a^	Multivariable Model 1 (Entered Variables)^b^	Multivariable Model 2 (Stepwise Selection)^c^
	HSV-2 Seroconverters (N = 614)	HSV-2 Non seroconverters (N = 3284)	IRR (95% CI); *P* Value	IRR (95% CI); *P* Value	IRR (95% CI); *P* Value
Baseline characteristics					
Age, y					
16–24	489 (79.6%)	2426 (73.9%)	Ref	Ref	
25+	125 (20.4%)	858 (26.1%)	0.74 (0.60–0.89), *P* = .002	0.93 (0.75–1.15), *P* = .503	
Marital status					
Never married	547 (89.1%)	2591 (78.9%)	Ref	Ref	
Married	66 (10.7%)	678 (20.6%)	0.61 (0.44–0.84), *P* = .003	1.05 (0.68–1.61), *P* = .824	
Previously married	1 (0.2%)	15 (0.5%)	0.43 (0.06–3.12), *P* = .403	0.36 (0.05–2.79), *P* = .329	
Lives with partner	99 (16.1%)	958 (29.2%)	0.57 (0.45–0.73), *P* < .001	0.64 (0.46–0.88), *P* = .006	0.64 (0.49–0.82), *P* = .001
Education					
No schooling/primary school	34 (5.5%)	300 (9.1%)	Ref		
Secondary school	466 (75.9%)	2411 (73.4%)	0.87 (0.55–1.37), *P* = .538		
Postsecondary school	114 (18.6%)	573 (17.4%)	0.87 (0.54–1.41), *P* = .565		
Any previous pregnancy	433 (70.5%)	2521 (76.8%)	0.83 (0.69–0.99), *P* = .036	0.90 (0.74–1.08), *P* = .251	
Multiple partners, prior 3 mo	54 (8.8%)	160 (4.9%)	1.59 (1.21–2.10), *P* = .001	1.23 (0.87–1.75), *P* = .243	1.29 (0.96–1.71), *P* = .083
New sex partner, prior 3 mo	35 (5.7%)	114 (3.5%)	1.45 (1.03–2.03), *P* = .033	1.07 (0.72–1.60), *P* = .737	
Number of vaginal sex acts, prior 3 mo Median (Q1, Q3)	7.0 (3.0, 15.0)	8.0 (3.0, 20.0)	1.00 (0.99–1.00), *P* = .614		
Any condomless sex, prior 3 mo	449 (73.1%)	2318 (70.6%)	1.14 (0.95–1.36), *P* = .149	1.10 (0.92–1.32), *P* = .314	
No condom last vaginal sex	276 (45.0%)	1507 (45.9%)	0.97 (0.82–1.13), *P* = .660		
Sex for money or gifts, prior 3 mo	7 (1.1%)	24 (0.7%)	1.48 (0.70–3.13), *P* = .301		
*Chlamydia trachomatis* infection	148 (24.1%)	557 (17.0%)	1.40 (1.16–1.69), *P* < .001	1.17 (0.96–1.43), *P* = .118	1.20 (0.98–1.45), *P* = .070
*Neisseria gonorrhoeae* infection	43 (7.0%)	104 (3.2%)	2.00 (1.47–2.72), *P* < .001	1.43 (1.04–1.97), *P* = .029	1.44 (1.03–1.97), *P* = .026
Follow-up characteristics					
Any pregnancy during follow-up	21 (3.4%)	92 (2.8%)	1.24 (0.80–1.91), *P* = .332		
Any new partner during follow-up	120 (19.5%)	407 (12.4%)	1.43 (1.17–1.74), *P* = .001	1.13 (0.83–1.54), *P* = .456	1.21 (0.97–1.48), *P* = .080
Any report of multiple partners during follow-up	115 (18.7%)	385 (11.7%)	1.43 (1.16–1.75), *P* = .001	1.10 (0.80–1.52), *P* = .547	
Any sex for money or gifts during follow-up	17 (2.8%)	69 (2.1%)	1.34 (0.83–2.17), *P* = .236		
HIV seroconversion during follow-up	90 (14.7%)	79 (2.4%)	4.41 (3.57–5.44), *P* < .001	3.58 (2.84–4.50), *P* = .000	3.55 (2.78–4.48), *P* < .001
*Chlamydia trachomatis* infection	11 (1.8%)	48 (1.5%)	1.71 (1.43–2.05), *P* < .001	1.38 (1.14–1.68), *P* = .001	1.40 (1.15–1.69), *P* = .001
*Neisseria gonorrhoeae* infection	8 (1.3%)	10 (0.3%)	2.38 (1.82–3.12), *P* < .001	1.86 (1.40–2.47), *P* = .000	1.87 (1.40–2.45), *P* < .001

Of note, there was correlation between marriage and cohabitation with partner, having a new partner and multiple sex partners during follow-up, and *C. trachomatis* and *N. gonorrhoeae* at baseline and final study visits, but these correlations were determined not to be strong enough to exclude from the models based on criteria of a correlation coefficient (*r*) > 0.80 or VIF > 5.

Abbreviations: CI, confidence interval; HIV, human immunodeficiency virus; HSV-2, herpes simplex virus type 2; IRR, incidence rate ratio; Q, quartile; Ref, reference.

Adjusted only for contraceptive method assigned at enrollment and country.

Adjusted for contraceptive method assigned at enrollment, country, and for all other variables with *P* < .20 in minimally adjusted analyses.

Stepwise model selection was used, which removes variables and selects the best model based on the Akaike information criterion. All variables with *P* < .20 in minimally adjusted analysis were included in stepwise selection model. Study arm and country were entered into the model.

## DISCUSSION

In this prospective, randomized study of young African women seeking contraceptive services, we found that HSV-2 incidence was high, with 1 in 8 acquiring the infection per year. DMPA-IM, which had been associated with elevated HSV-2 risk in some prior observational studies, did not have elevated HSV-2 incidence compared with the other 2 methods.

There are limited published data assessing whether contraceptive method influences acquisition of HSV-2. Available studies have been observational and have focused on the association between DMPA-IM use and HSV-2 infection, with very limited data assessing risk for users of LNG implants or copper IUDs. Two recent systematic reviews identified 3 studies that reported increased incidence of HSV-2 with DMPA-IM use [17, 18]. One, from Uganda, found a 2-fold increased risk of HSV-2 infection with use of DMPA compared to noncontraceptive users. The results were dependent on 9 incident HSV-2 infections among 57 women reporting consistent DMPA-IM use [9]. The second, from Canada, found a 4-fold increase risk with use of DMPA-IM among female sex workers, but this study had only 19 consistent DMPA-IM users who had marked differences in sexual risk behavior compared to noncontraceptive users [8]. The third, from Rwanda, found a 2-fold increase risk of HSV-2 infection with DMPA use among female sex workers; these findings were based on data from 26 consistent DMPA users [8]. Thus, these 3 studies had significant limitations, including very few women with consistent DMPA-IM use, exposure ascertainment only by self-report, and comparator groups including women who were not pregnant and not using any contraceptive, which could introduce bias because of differences in sexual risk behavior. Our findings concur with results from 2 studies evaluating African women engaged in transactional sex and did not find an association between DMPA-IM use and HSV-2 incident infection [11, 19] and an additional study from HIV serodifferent couples in east and southern Africa [12].

We found that DMPA-IM users had a significantly lower incidence of HSV-2 compared with copper IUD users but not compared with LNG implant users. Copper IUD and LNG implant users did not have statistically significant differences in HSV-2 incidence. Our data could suggest some increased HSV-2 risk for the copper IUD, at least compared with DMPA-IM, but the range in possible values of the IRR was compatible with as low as a 3% difference in relative risk. Users of all 3 methods of contraceptive had high HSV-2 incidence, similar to findings from other studies with no contraceptive users. Given the differences in contraceptive mechanism of action of the copper IUD and the hormonal contraceptives, it does not seem plausible that all the methods orchestrated increased risk for HSV-2 infection.

The high HSV-2 incidence we observed, aligned with the high detection of *C trachomatis* and *N gonorrhoeae* seen in this clinical trial population [20], as well as with the high HIV incidence. Together, those data reflect high risk of HIV and other STIs among adolescent girls and young women in East and Southern Africa seen across multiple studies [21, 22]{Torrone, 2018 #142). Infection with *N gonorrhoeae*, *C trachomatis*, and incident HIV infection were associated with increased HSV-2 acquisition, all consistent with expectations. The synergy between HIV acquisition and HSV-2 infection is well documented and our data provide additional validation of this relationship [23]. Our data have limitations. HSV-2 infection was assessed at only 2 time points for each woman, and we therefore cannot determine the sequence of events in assessing for association in HSV-2 risk with diagnosis of STIs such as HIV, *C trachomatis*, and *N gonorrhoeae* infection.

In comparison to previously published studies, our study represents data from the largest prospective cohort of young African women to date with 3898 HIV/HSV-2 negative women and 614 incident HSV-2 infections, evaluated for risk of HSV-2 with use of 3 commonly used contraceptives and tested using a randomized trial design. Our findings are strengthened by the high quality of study conduct, with successful randomization to 3 highly effective contraceptive methods, rigorous ascertainment of exposure (contraceptive use) and outcome (HSV-2), and > 90% adherence to use of randomly assigned contraceptive method and retention to study visits [13]. The randomized design reduces bias compared with observational studies, where a woman’s choice of contraceptive methods may reflect differences in sexual risk behavior, sociodemographic differences, or other factors.

## CONCLUSION

Our data provide reassurance that contraceptive method, especially DMPA-IM, is not a strong risk factor for acquisition of HSV-2. These findings complement the primary HIV acquisition results of this trial [13], thus favoring global efforts to expand access to highly effective contraceptives such as DMPA-IM, copper IUD, and the LNG implant.

## Supplementary Data

Supplementary materials are available at *Clinical Infectious Diseases* online. Consisting of data provided by the authors to benefit the reader, the posted materials are not copyedited and are the sole responsibility of the authors, so questions or comments should be addressed to the corresponding author.

ECHO Trial Consortium

Management Committee:

Jared M. Baeten (University of Washington, Seattle, Washington, USA), James Kiarie (WHO, Geneva, Switzerland), Timothy D. Mastro (FHI 360, Durham, North Carolina, USA), Nelly R. Mugo (Kenya Medical Research Institute, Nairobi, Kenya; University of Washington, Seattle, Washington, USA), Helen Rees (Wits Reproductive Health and HIV Institute, Johannesburg, South Africa).

Study Site Principal Investigators:

Manzini, Eswatini: Jessica Justman, Zelda Nhlabatsi (Family Life Association of Eswatini & ICAP at Columbia University, New York, New York, USA). Kisumu, Kenya: Elizabeth A. Bukusi, Maricianah Onono (Kenya Medical Research Institute, Nairobi, Kenya). Brits, South Africa: Cheryl Louw (Madibeng Centre for Research). Cape Town, South Africa: Linda-Gail Bekker, Gonasagrie Nair (University of Cape Town and Desmond Tutu HIV Centre). Durban, South Africa: Mags Beksinska, Jennifer Smit (MatCH Research Unit, Faculty of Health Sciences, University of the Witwatersrand). East London, South Africa: G. Justus Hofmeyr, Mandisa Singata-Madliki (University of Fort Hare and University of the Witwatersrand). Edendale: South Africa: Jennifer Smit (MatCH Research Unit, Faculty of Health Sciences, University of the Witwatersrand). Johannesburg, South Africa: Thesla Palanee-Phillips (Wits Reproductive Health and HIV Institute, Faculty of Health Sciences, University of the Witwatersrand). Klerksdorp, South Africa: Raesibe Agnes Pearl Selepe (The Aurum Institute). Ladysmith, South Africa: Sydney Sibiya (Qhakaza Mbokodo Research Clinic). Soshanguve, South Africa: Khatija Ahmed (Setshaba Research Centre). Lusaka, Zambia: Margaret Phiri Kasaro, Jeffrey Stringer (UNC Global Projects Zambia and University of North Carolina at Chapel Hill, Chapel Hill, North Carolina, USA).

Other members of the ECHO Trial Consortium:

Deborah Baron (Wits Reproductive Health and HIV Institute, Faculty of Health Sciences, University of the Witwatersrand, Johannesburg, South Africa), Deborah Donnell (University of Washington and Fred Hutchinson Cancer Research Center, Seattle, Washington, USA), Peter B. Gichangi (International Centre for Reproductive Health—Kenya & Technical University of Mombasa, Mombasa, Kenya), Kate B. Heller (University of Washington, Seattle, Washington, USA), Nomthandazo Mbandazayo (Wits Reproductive Health and HIV Institute, Johannesburg, South Africa), Charles S. Morrison (FHI 360, Durham, North Carolina, USA), Kavita Nanda (FHI 360, Durham, North Carolina, USA), Melanie Pleaner (Wits Reproductive Health and HIV Institute, Faculty of Health Sciences, University of the Witwatersrand, Johannesburg, South Africa), Caitlin W. Scoville (University of Washington, Seattle, Washington, USA), Kathleen Shears (FHI 360, Washington, DC, USA), Petrus S. Steyn (WHO, Geneva, Switzerland), Douglas Taylor (FHI 360, Durham, North Carolina, USA), Katherine K. Thomas (University of Washington, Seattle, Washington, USA), Julia D. Welch (FHI 360, Durham, North Carolina, USA).

ciab1027_suppl_Supplementary_Figure_S1

## Notes

***Author Contributions***. J. M. B., D. D., and N. R. M. designed the study. R. S. and D. D. performed the data analyses and approved the final results interpretation. N. M., C. S., C. M., D. D., H. R., J. R., J. D., M. B., N. P., E. B., I. B., V. E., A. K., and R.H. contributed to the execution of the trial, and all authors reviewed and approved the final manuscript.

***Acknowledgments.*** The authors thank the women who participated in this study for their motivation and dedication, and the different personnel at the 12 clinical trial teams for meticulous trial conduct. The conduct of this trial involved the engagement of a large community of global and local site partners. Data management was done by DF/Net, Inc. (Seattle, Washington, USA); laboratory support was done by Clinical Laboratory Services, a division of the Wits Health Consortium of the University of Witwatersrand’s School of Pathology (Johannesburg, South Africa).

***Disclaimer.*** The study funders and manufacturers of the trial contraceptive methods were not involved in the execution of the trial, interpretation of results, or decision to submit for publication. The authors designed the study, gathered the data, performed all analyses, vouch for the data completeness, prepared the manuscript, and were responsible for the decision to submit for publication.

***Financial support.*** The Evidence for Contraceptive Options and HIV Outcomes (ECHO) Trial was supported by the combined generous support of the Bill & Melinda Gates Foundation (grant OOPP1032115), the American people through the United States Agency for International Development and the President’s Emergency Plan for AIDS Relief (grant AID-OAA-A-15-00045), the Swedish International Development Cooperation Agency as part of the EDCTP2 programme supported by the European Union (grant 2017/762965-0), the South African Medical Research Council, and the United Nations Population Fund. Contraceptive supplies were donated by the Government of South Africa and United States Agency for International Development. Additional funding for testing for herpes simplex type 2 infection was provided in part by the US Centers for Disease Control and Prevention (U48 DP 005013 SIP 14-023) and the University of Washington/Fred Hutch Center for AIDS Research (US National Institutes of Health P30 AI057757). The contents of this paper are solely the responsibility of the authors and do not necessarily reflect the views, decisions, or policies of the institutions with which they are affiliated, the ECHO trial funders, or the supporting governments.
