# Supplementary figures and images for: Incidence of Herpes Simplex Virus Type 2 Infection Among African Women Using Depot Medroxyprogesterone Acetate, a Copper Intrauterine Device, or a Levonorgestrel Implant for Contraception: A Nested Randomized Trial
Source: Clin Infect Dis. 2021 Dec 15;75(4):586–95. doi: 10.1093/cid/ciab1027 (PMC9464069; doi:10.1093/cid/ciab1027)

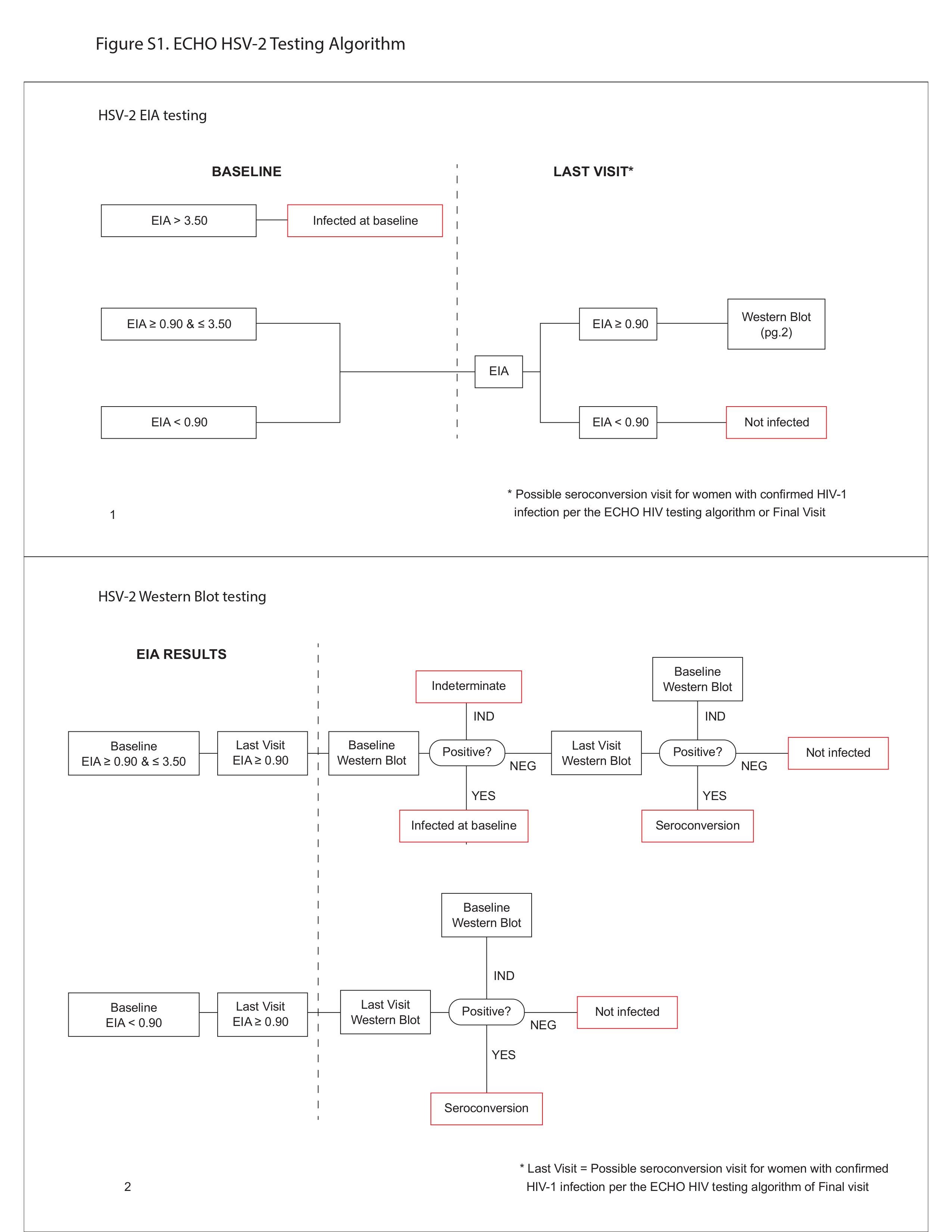

Supplement: ciab1027_suppl_Supplementary_Figure_S1 [file ciab1027_suppl_supplementary_figure_s1.jpeg]
